# FASSVid: Fast and Accurate Semantic Segmentation for Video Sequences

**DOI:** 10.3390/e24070942

**Published:** 2022-07-07

**Authors:** Jose Portillo-Portillo, Gabriel Sanchez-Perez, Linda K. Toscano-Medina, Aldo Hernandez-Suarez, Jesus Olivares-Mercado, Hector Perez-Meana, Pablo Velarde-Alvarado, Ana Lucila Sandoval Orozco, Luis Javier García Villalba

**Affiliations:** 1 Instituto Politecnico Nacional, ESIME Culhuacan, Mexico City 04440, Mexico; jportillop@ipn.mx (J.P.-P.); gasanchezp@ipn.mx (G.S.-P.); ltoscano@ipn.mx (L.K.T.-M.); alhernandezsu@ipn.mx (A.H.-S.); jolivares@ipn.mx (J.O.-M.); hmperezm@ipn.mx (H.P.-M.); 2 Unidad Académica de Ciencias Básicas e Ingenierías, Universidad Autónoma de Nayarit, Tepic 63000, Mexico; pvelarde@uan.edu.mx; 3 Group of Analysis, Security and Systems (GASS), Department of Software Engineering and Artificial Intelligence (DISIA), Faculty of Computer Science and Engineering, Office 431, Universidad Complutense de Madrid (UCM), 28040 Madrid, Spain; asandov@ucm.es

**Keywords:** semantic segmentation, real-time processing, semantic video segmentation, embedded systems

## Abstract

Most of the methods for real-time semantic segmentation do not take into account temporal information when working with video sequences. This is counter-intuitive in real-world scenarios where the main application of such methods is, precisely, being able to process frame sequences as quickly and accurately as possible. In this paper, we address this problem by exploiting the temporal information provided by previous frames of the video stream. Our method leverages a previous input frame as well as the previous output of the network to enhance the prediction accuracy of the current input frame. We develop a module that obtains feature maps rich in change information. Additionally, we incorporate the previous output of the network into all the decoder stages as a way of increasing the attention given to relevant features. Finally, to properly train and evaluate our methods, we introduce CityscapesVid, a dataset specifically designed to benchmark semantic video segmentation networks. Our proposed network, entitled FASSVid improves the mIoU accuracy performance over a standard non-sequential baseline model. Moreover, FASSVid obtains state-of-the-art inference speed and competitive mIoU results compared to other state-of-the-art lightweight networks, with significantly lower number of computations. Specifically, we obtain 71% of mIoU in our CityscapesVid dataset, running at 114.9 FPS on a single NVIDIA GTX 1080Ti and 31 FPS on the NVIDIA Jetson Nano embedded board with images of size 1024×2048 and 512×1024, respectively.

## 1. Introduction

Semantic segmentation is considered as one of the fundamental tasks in computer vision along with tasks such as image classification, object detection and instance segmentation [1,2,3,4]. Its purpose is to assign class labels to each pixel in a given input image. In recent years, with the rise of new deep learning techniques, semantic segmentation has been successfully applied to several challenging fields, for instance: autonomous driving, medical imaging, augmented reality, and remote sensing, to mention a few [5]. Some important semantic segmentation research has specific applications, such as that proposed in [6], which presents a framework to create foreground–background masks from depth images for human body segmentation. In [7], a fully connected VGG16 neural network was proposed for real-time path finding intended to help visually impaired or blind people. In [8], a novel neural-network model called F^2^2CNN which integrates a feedback mechanism into deep FCNN was proposed for outdoor video semantic segmentation.

As a dense prediction task, semantic segmentation requires a lot of computational operations in order to produce accurate results. For non-real-time applications, this usually does not represent a major problem. However, for certain applications, such as autonomous driving cars, speed and accuracy are required to rapidly and reliably take actions when needed [3,9]. The problem is that speed and accuracy are two factors that contradict each other, making the real-time implementation a challenging problem [2]. Moreover, if implemented on embedded devices, low energy consumption, and efficient memory usage also become crucial factors, which adds an extra layer of complexity on top of the already difficult task [10].

The vast majority of methods for real-time semantic segmentation are designed with the idea of performing per-frame inference as quickly as possible [3,10,11,12]. However, they do not take into consideration the temporal information present in frames of the video sequences. We believe that this is counter-intuitive for real-world applications where the main application of such real-time methods is, precisely, being able to process frame sequences as quickly as possible.

On the other hand, there are some methods that consider the temporal information available intrinsically in the video sequences [13,14,15,16]. They do so by extracting clues from previous frames or through information from other domains such as motion detection masks, optical flow, depth maps or even vehicle odometry. However, most of these methods are extremely slow to compute, making them unfeasible for real-time implementation.

We address the aforementioned issues by developing a fast and accurate network that exploits the temporal information present in previous states of the video stream. First, we develop FDChangeNet, a sub-network specifically designed for handling the pair of input frames [$I_{t}$, $I_{t-k}$] of the sequence and extract the “change” information between them. We then use this information and fuse it with rich contextual features coming from a feature extraction network enhanced with our custom spatial pyramidal module. We further increase the accuracy of the network by developing the TAM module which incorporates the previous output of the network $O_{t-1}$ into our lightweight decoder, thus enabling a temporal attention mechanism in the network.

Furthermore, following our video frame sequence approach, we also propose a methodology for training and evaluating semantic video segmentation networks. To this end, we introduce a new dataset based on the Cityscapes video data [17]. Since there are no official labels for each of the frames in the sequence, we obtain their respective pseudo-groundtruth employing FC-HarDNet-70 [11], an open-source high-accuracy network already trained in *Cityscapes Fine* [17]. We then split the data into six video frame sequences in a 70:30 (training:testing) fashion. Finally, we trained our network with sequential input batches as opposed to the standard random batch sampling used in per-frame training approaches.

**Figure 1:**
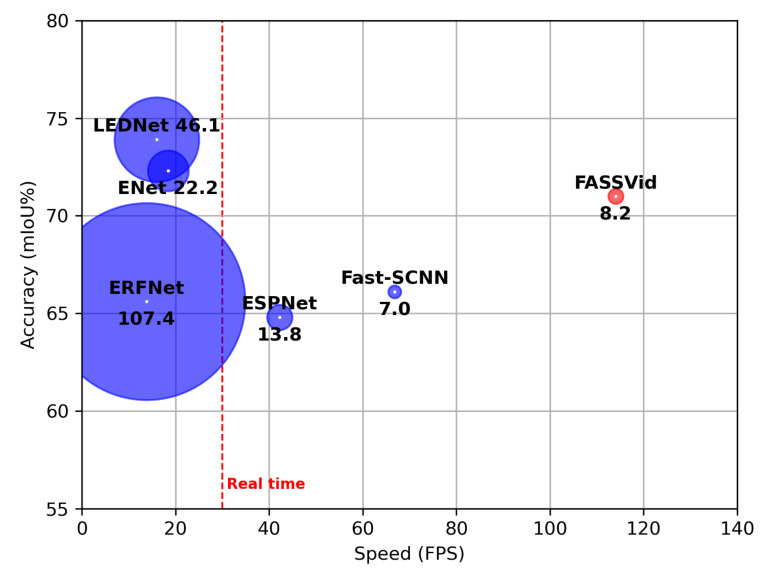
Speed and accuracy comparison between our proposal FASSVid and other lightweight networks on the *CityscapesVid* dataset. The speed was measured on an NVIDIA GTX 1080Ti. The number alongside the model indicates the required number of computations in terms of GFLOPs per tensor of size 1×3×1024×2048.

To summarize, our contributions can be listed as follows:

- We propose a methodology for evaluating the performance of semantic video segmentation networks and introduce *CityscapesVid*, a new dataset based on Cityscapes.
- We introduce *FASSVid*, a fast and accurate lightweight network designed specifically for semantic video segmentation. As shown in Figure 1, FASSVid surpasses the speed of all other state-of-the-art lightweight networks, maintaining high accuracy and a low number of computations.
- We demonstrate the effectiveness of our methods through multiple experiments and report the implementation results on the NVIDIA GTX 1080Ti GPU and the NVIDIA Jetson Nano embedded board.
- This proposal is an evolution of a previous work of the authors, FASSDNet; both works share a common structure; however, FASSVid, is intended to obtain information from a sequence of frames using temporal information, while FASSDNet aims at segmentation in isolated frames.

## 2. Related Work

### 2.1. Semantic Segmentation

In the literature, methods based on Fully Convolutional Networks (FCNs) [18] have achieved remarkable performance on several semantic segmentation benchmarks [19]. Nowadays, FCNs are the dominant methods for performing semantic segmentation [19,20,21,22]. There have been several proposals to enhance the prediction accuracy. The most common way to achieve this goal is leveraging the use of encoder–decoder architectures [12,23], for example, the use of HarDNet architecture [11], to achieve high efficiency in terms of both low MACs and memory traffic, and the use of compact encoders and lightweight decoders [19], but in general this consists of: (1) an encoder block that gradually downsamples and learns feature maps with high semantic information, and (2) a decoder block that recovers the spatial information [19]. Notably, there are *U-Net*-like networks that incorporate skip connections between the encoder and decoder in order to recover spatial information present in early stages of the network. In addition, there are also methods for extracting feature maps rich in contextual information. Methods that exploit multi-scale information such as SPP [21] and ASPP [19] perform a series of convolutions in the same set of feature maps at multiple scales, whereas methods such as [1,3,24] learn attention masks that focus on relevant information.

Our proposed network leverages the standard U-Net shape architecture and incorporates a pyramidal pooling module to increase the prediction accuracy. Specifically, our network employs a custom version of the ASPP module [19] modified in such a way that the number of floating-point operations, and by extension, the number of parameters required by the network, is reduced to a large extent.

### 2.2. Real-Time Semantic Segmentation

High-performance semantic segmentation networks, although very accurate, are expensive to compute even in modern GPUs, making them unfeasible for real-time applications [25]. Techniques such as depthwise separable convolutions [26,27,28,29], zoomed convolutions [30] or convolution factorization [10,25,31] have been proposed in other works to address this issue. However, networks that employ these techniques achieve real-time performance at the cost of significantly lower accuracy.

Lightweight real-time networks such as ESPNet [32,33], ENet [34] and LEDNet [35] employ lightweight pyramidal multi-resolution strategies similar to DeepLabV3+ [19] in order to increase the segmentation accuracy. Specifically, LEDNet [35] and ESNet [31] incorporate asymmetric convolutions in their core modules, which reduces both the number of parameters and the number of computations. However, they strongly rely on these strategies, negatively affecting the speed performance, compared to highly optimized standard convolutions available in deep learning frameworks [12].

Contrary to this type of network, our work is focused on increasing the semantic segmentation accuracy in video frame sequences with as fast inference speed as possible. To this end, we carefully designed our modules to maintain an appropriate trade-off between speed, accuracy and “lightweightness” in terms of number of parameters and floating-point operations. Figure 1 shows a comparison of speed, accuracy and the number of operations required in terms of GFLOPs, between our proposed model FASSVid and other lightweight artificial neural networks.

### 2.3. Video Segmentation

Methods designed specifically for semantic video segmentation have been proposed in order to exploit temporal information and to incorporate other clues whenever possible [13,14,15,16]. In practice, the majority of methods that deal with video sequences fail to be executed with real-time performance, since it is not usually their main goal. Instead, they tend to focus on either increasing the accuracy performance or reducing temporal redundancies (i.e., keeping a consistent segmentation during the entire video sequence) [16,36,37], also in a dynamic video segmentation network (DVSNet) [13], and in the use of temporal consistency in the inference phase [14]. The most common approaches to incorporating clues are through information from other domains. Normally, this is done with *multi-stream architectures*, where multiple inputs such as previous frames of que sequence [13,14,36] or the optical flow maps [15,38,39] are fed into the network. These inputs are then processed by the network to produce more accurate predictions. There are now recent and important works, such as Shelhamer et al. [40] who proposed a network named Clockworks, which is a combination of FCN and the clockwork recurrent network, grouping the layers of the network into stages with different rates (either fixed clock rate or adaptive clock) and then fusing them via skip connections. Hu et al. [41] proposed TDNet, a network for video semantic segmentation, by circularly distributing sub-networks over sequential frames, leveraging temporal continuity. Currently, a main disadvantage of the semantic segmentation methods to process videos is performing individual processing of each frame; as has been explained, this approach disregards the temporal information that exists in the video sequences; a potential solution is the use of recurrent neural networks, which take into account temporal information; traditionally, a convolutional LSTM between encoder and decoder layers has been used; however, nowadays we do not have classical encoder/decoder structures, but rather multi-branch architectures that enable an extension of their capabilities by placing convolutional LSTM layers in different positions of the CNN networks, which have been shown to improve performance [42].

Our proposed network can also be seen as a multi-stream architecture since it incorporates a sub-network that exploits the temporal information between two input frames. However, our network does this on the fly, as opposed to similar approaches [38,39] where it is assumed that the input optical flow maps are already pre-computed. This results in our method being faster if implemented in real-world autonomous driving scenarios.

## 3. Proposed Methodology

### 3.1. Network Architecture

Virtually all high-performance state-of-the-art methods for semantic segmentation, such as [1,23,24], are built on top of other high-performance networks for image classification, such as ResNet [43], Wide-ResNet [44] or Xception [28]. We adopt a custom version of HarDNet [11] as our backbone. We choose HarDNet because of its powerful encoding capabilities and reduced number of parameters. Moreover, HarDNet is optimized to reduce the Dynamic Random-access Memory (DRAM) traffic in the GPU, leading to a boost in inference speed, since less accesses to the memory need to be made.

In our custom HarDNet implementation, we remove the last encoding block from the original version and replace it with our Custom ASPP module (C-ASPP) to enhance the multi-scale feature representation. In addition, we set the convolution stride to 2 in the third convolutional layer of the network to reduce by half the size of the feature maps, thus reducing the computational burden in subsequent stages.

**Figure 2:**
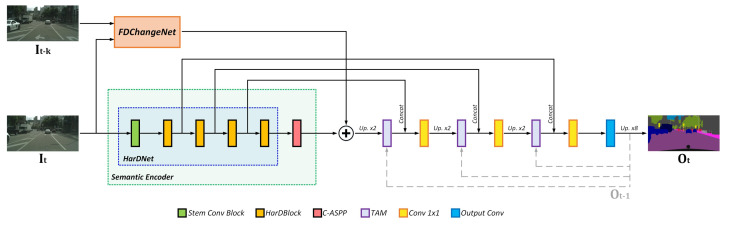
Proposed network architecture.

We perform the fusion of features coming from our sub-network FDChangeNet and our proposed HarDNet + C-ASPP through addition. Then, we upscale and refine the resulting feature maps through all the decoder stages. We incorporate the skip connections coming from the encoder stages into their respective stage in the decoder. On top of that, we also incorporate the resulting feature maps from the previous output of the network into the decoding process. Finally, the last stage of our network consists of a single 1×1 convolution for making the final prediction. Bilinear interpolation is used to reestablish the original input size (1024×2048). The resulting network is shown in Figure 2.

#### 3.1.1. Custom Atrous Spatial Pyramidal Pooling Module (C-ASPP)

The ASPP module was originally proposed in DeepLab [45] to robustly segment objects at multiple scales. The main idea of ASPP is that convolutions with multiple receptive fields capture objects as well as image context at multiple scales. The ASPP module is based on standard atrous (dilated) convolutions within five pyramidal branches. Specifically:

- 1 × Conv 1 × 1
- 1 × Pooling + Conv 1 × 1
- 3 × Atrous Conv 3 × 3 (with dilation rates r = 12, 24 and 36, respectively).

**Figure 3:**
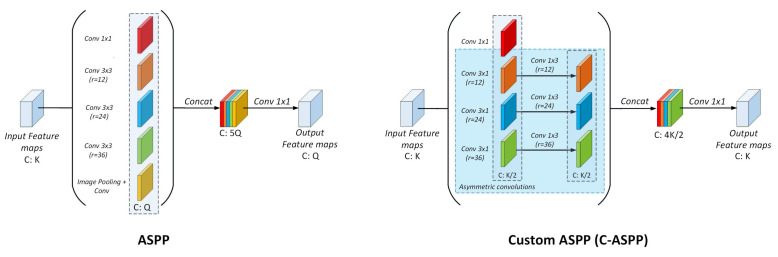
Pyramidal method comparison. **Left**: Original ASPP. **Right**: Our Custom ASPP implementation (C-ASPP).

In order to reduce the computational burden, in our custom ASPP implementation we factorize the 2D 3 × 3 atrous convolutions into two consecutive 1D atrous convolutions. Specifically, each of the original convolutions becomes a 3 × 1 convolution followed by a 1 × 3 convolution. We refer to this type of convolution as an asymmetric convolution, due to the nature of the asymmetrical-shape convolution kernels. In addition, we also remove the image pooling branch since it computes feature maps that are likely to be learned through the 1 × 1 convolutional branch, saving more computations in the process. Finally, the number of output feature maps of each pyramidal branch changes dynamically depending on the number of input feature maps of the module. Specifically, the number of output feature maps *Q* of each branch is defined by *K* × $\frac{1}{\alpha }$, where *K* is the number of input feature maps and α serves as the compression factor. In our experiments, we set α=2. Figure 3 illustrates the differences between the original ASPP and our custom implementation. Following the proposed asymmetric strategy with α=2, our C-ASPP performs $\left(K\times d\times \frac{1}{2}K\right)+\left(\frac{1}{2}K\times d\times \frac{1}{2}K\right)$ operations. To put this in perspective, for a given 3 × 3 kernel, our factorization strategy requires $\frac{1}{2}$ of the original number of operations of ASPP, thus saving 50% of the needed computations.

#### 3.1.2. FDChangeNet

Our proposed sub-network FDChangeNet takes two video frames $I_{t}$ and $I_{t-k}$ as inputs. Specifically, $I_{t}$ is the current frame of the sequence and $I_{t-k}$ is the respective *k*-th previous frame; frames between *t* and t−k are not used at this stage of the processing. The parameter *k* allows us to select the temporal distance between frames of the video sequence; if *k* is relatively small, the changes between images are very little; on the other hand, if *k* is relatively big, the changes between images are bigger; it is worth mentioning that *k* would depend on the FPS. In our experiments, we set k=10 (unless specified otherwise) because in typical day-to-day applications around 25 FPS, k=10 gives us the necessary results to validate our proposal.

**Figure 4:**
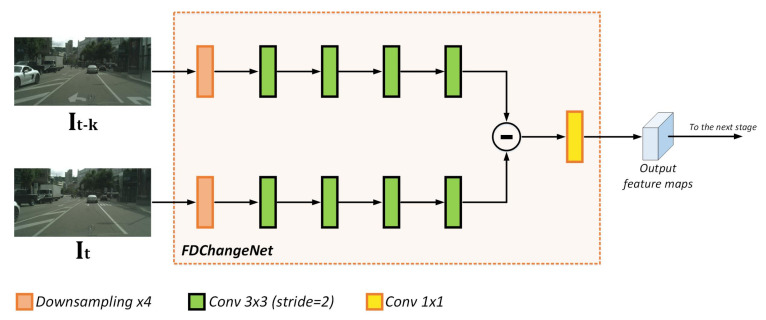
FDChangeNet architecture.

As shown in Figure 4, FDChangeNet consists of two convolutional branches that encode the input frame information. Although these branches are the same, the weights are independent of each other. Since the purpose of this module is to obtain the change information rather than contextual information, we first downsample the pair of frames down to $\frac{1}{4}$ of the original image size, greatly reducing the computational burden. Then, each convolutional branch processes the input frames through a series of 3×3 convolution layers with stride=2. In the following, whenever the convolution operation is mentioned, it is referred to as the composite operation conv+BatchNorm+ReLU. The resulting feature maps are subtracted and further processed by a 1×1 convolution for the next stage. With this module, we extract the change information between two frames, so that the decoding process focuses on relevant regions that change with respect to the k-th previous frame.

#### 3.1.3. Temporal Attention Module (TAM)

**Figure 5:**
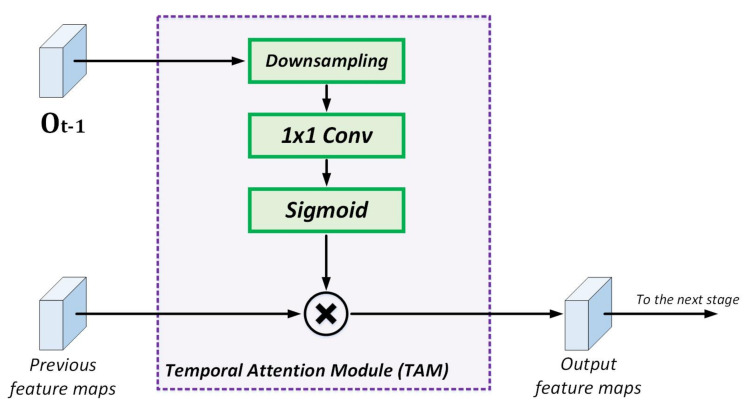
Temporal Attention Module (TAM).

As a way to increase the attention to relevant features in the video sequence, we incorporate the previous output of the network $O_{t-1}$ into the decoding process of the currently processed frame $I_{t}$; for the initialization stage, $O_{t-1}$ are set to 0 (image of 0’s), since in time t=0 the previous frames do not exist. The tensor $O_{t-1}$ of size 1×C×H×W (batch_size × number of classes × height × weight) is downsampled to $\frac{1}{16}$, $\frac{1}{8}$ and $\frac{1}{4}$ of its original size, depending on the decoder stage. Similarly, in each TAM module, a convolution layer adjusts the number of channels needed by the attention step. As shown in Figure 5, TAM comprises a sigmoid weighted multiplication between the input of each decoder stage and the downsampled $O_{t-1}$. Mathematically, this can be described as:

(1) $$Y_{l}=\sigma \left(F\left(O_{t-1}\right)\right)*X_{l}$$

where $X_{l}$ and $Y_{l}$ represent the input and output feature maps of the attention process at each decoder stage *l*; $F(O_{t-1})$ is the previous output of the network after the downsampling and convolution process; and σ denotes the sigmoid operation that squeezes the values in $O_{t-1}$ down to the closed range [0–1].

### 3.2. Sequential Training and Evaluation for Semantic Video Segmentation

In an effort to evaluate semantic video segmentation, the Cityscapes dataset [17] has available 500 30-frame snippets recorded at 17 Hz (upon request by e-mail). However, the time for each snippet is equivalent to 1.76 s of video, which is insufficient for long-term evaluation of semantic video segmentation methods. On top of that, each snippet contains only one groundtruth image, making the training of these methods a challenging problem since very little data is available for supervised learning approaches.

Cityscapes provides the frames of three video sequences exclusively for qualitative evaluation of computer vision tasks. Because of this, these video sequences, namely *stuttgart_00*, *stuttgart_01* and *stuttgart_02*, do not include annotations (labels) of any kind.

In order to train and evaluate our proposed networks, we generate the semantic labels for each frame of the three video sequences using FC-HarDNet-70 [11]. We refer to these labels as the pseudo-groundtruth since they are not exact annotations made by humans, but instead they are obtained through a high-performance semantic segmentation network. Our proposed dataset, *CityscapesVid*, is composed of 2899 input frames with their respective groundtruth. It is split into two sets: the train and test sets. Due to the limited number of frames in the sequences, and for the sake of a long-term evaluation (i.e., extended video length), we omit the validation set and instead evaluate our proposals directly on the test set.

The train and test sets are distributed in a 70:30 fashion: 70% of the data for training and the remaining 30% for testing. Each video sequence is divided following this principle. Specifically, for each video sequence:

- *stuttgart_00*: Frames from 1 to 420 are for training and frames from 421 to 599 are for testing.
- *stuttgart_01*: Frames from 1 to 770 are for training and frames from 771 to 1100 are for testing.
- *stuttgart_02*: Frames from 1 to 840 are for training and frames from 841 to 1200 are for testing.

**Figure 6:**
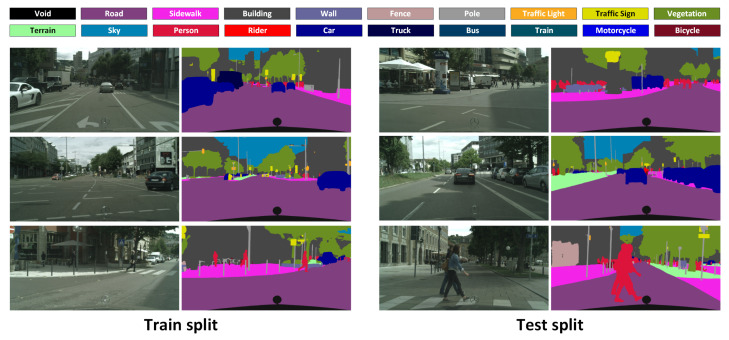
Examples inputs and their respective pseudo-groundtruths of our proposed dataset splits.

The proposed video split results into three video sequences for training and three sequences for testing. Some example images of the proposed splits are shown in Figure 6.

**Figure 7:**
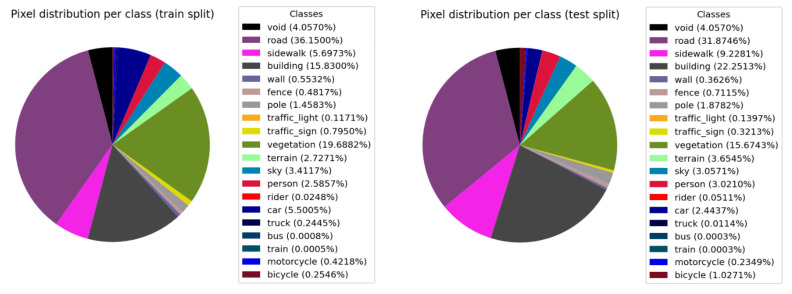
Perclass distribution of the proposed video frame splits.

Details about the class distribution in the dataset are illustrated in Figure 7. In our experiments, we exclude the rider, truck, bus, train and void classes from the training and evaluation processes. The reason behind this is that the bus and train classes do not appear in the dataset. Similarly, the rider and truck classes, although they appear in a few frames of the sequences, most of the time do it as noise (due to the pseudo-groundtruth obtention process). Furthermore, these two classes represent only 0.07% and 0.25% of the complete dataset, respectively. As for the void class, following the convention of the Cityscapes Fine dataset, we designate it as the area corresponding to the hood of the car from where the camera is capturing the images. This area can be appreciated in the example groundtruths of Figure 6.

## 4. Results

The performance of the evaluated methods in terms of accuracy and inference speed is measured in mean Intersection over Union (mIoU) score (also known as mIoU accuracy) and frames per second (FPS). Additionally, We also report the number of parameters and the computational complexity (in GFLOPs). All experiments are performed on our proposed CityscapesVid dataset. For a fair comparison between networks, we evaluated the mIoU score, inference speed and computational complexity under images of size 1024×2048, which is the original image resolution of Cityscapes [17].

### 4.1. Experimental Setup

All models were trained on an Intel Core i9-9700K desktop with one NVIDIA RTX 2080Ti card and implemented in PyTorch 1.7. We used Stochastic Gradient Descent (SGD) with weight-decay $5\times 10^{-4}$ and momentum 0.9 as optimizer and employ the “poly” learning rate strategy $lr=initial\_lr\times {\left(\frac{iter}{total\_iter}\right)}^{0.9}$, with an initial learning rate of 0.02. Cross-entropy loss is computed following the online bootstrapping strategy [46]. Data augmentation consists of random horizontal flip, random scale in the range [0.5,2] and random cropping with 1024×1024 crop size. All the networks were trained with native PyTorch automatic mixed precision. We trained all models for 300 epochs with batch size 16. The same training setup is used for all models unless otherwise specified. For our final model, we follow the same training protocol but pre-train the model with *Cityscapes Fine* and fine-tune for 200 additional epochs. The inference speed in FPS of all methods is calculated from the average of 10,000 iterations run on an NVIDIA GTX 1080Ti card. Nevertheless, in order to make a fair comparison with those network models that used a GTX 1080Ti card to make inferences, we used an Intel i7-8700K + NVIDIA RTX 1080Ti only for inference purposes.

### 4.2. Ablation Study

We performed experiments for our two proposals, our sub-network DFChangeNet and our TAM module. We first experimented with the position of DFChangeNet. As shown in Table 1, when we establish the sub-network position after the C-ASPP module we obtain a 0.7% of mIoU improvement and a slight increase in FPS compared with the case where the sub-network is used before the C-ASPP. We believe that this is due to the strong semantic representation obtained by C-ASPP that complements well the temporal information extracted by DFChangeNet. On the other hand, when the temporal information is fused together with a still not-so-powerful semantic representation, the features rich in context may be compromised, thus resulting in C-ASPP not being able to exploit them properly.

After determining the best place to position our sub-network, we experimented with the number of filters per convolutional layer in DFChangeNet. To increase the inference speed of the network, we explored the case where the number of filters per layer is reduced in half with respect to the standard version obtained from the previous experiment. As shown in Table 2, the light version with half the number of filters per layer not only considerably reduces the mIoU accuracy with respect to the standard version, but also reduces it with respect to the baseline model. Specifically, the mIoU accuracy drops by 1.7% and 1% for each case, respectively. As a result, we determine that the small improvements in FPS and the computational complexity are not worth the loss of mIoU accuracy for the following experiments.

As for our TAM module, we conducted experiments to determine the best strategy and place to fuse the previous output $O_{k-1}$ within the decoder stages. In concrete, we experimented with four different setups:

1. *Baseline + Att:* Attention directly to the output convolution layer of the network with the raw $O_{k-1}$ (no Conv 1×1).
2. *Baseline + Att_conv:* Attention directly to the output convolution layer of the network ($O_{k-1}$ processed by a Conv 1×1).
3. *Baseline + Att_conv_all:* Attention to both every decoder stage as well as the output convolution layer of the network (the latter follows the same strategy as in “Baseline + Att”).
4. *Baseline + Att_conv_dec:* Attention to all the decoder stages only (as illustrated in Figure 2).

The results of these experiments are shown in Table 3. From the experiments, we observe that *Baseline + Att_conv* is the strategy that most dramatically decreases the speed performance in FPS compared with the baseline model. This is due to the convolution layer that has to process the set of feature maps $O_{k-1}$, which are at high resolution. On top of that, the mIoU accuracy drops by 0.6%, making this strategy even less effective than the baseline model. This is also the case for the *Baseline + Att* experiment, where the mIoU accuracy drops by the same amount, but with slightly less FPS degradation. Similarly, the *Baseline + Att_conv_all* strategy obtains the lowest result in terms of mIoU accuracy. We believe that this is due to the attention given to the output convolution layer where it is no longer needed, as demonstrated in the last experiment. The last experiment *Baseline + Att_conv_dec* exclusively uses the attention module in the decoder stages, deprecating all use of it in the output convolution layer. The results show that this is the optimal configuration strategy for our TAM module. We obtain a small increment of 0.5% of mIoU accuracy over the baseline with little negative effect in all other metrics.

Finally, our ablation study is summarized in Table 4. We observe how the two proposals by themselves do not much increase the accuracy performance in terms of mIoU. However, when they are combined, they achieve 2.4% mIoU accuracy improvement with respect to the baseline. The combination of our two proposals results in our final network architecture: *FASSDVid*.

### 4.3. Comparison with Other State-of-the-Art Lightweight Networks

We perform a comparative study between our proposal and the other five state-of-the art lightweight networks to demonstrate the effectiveness of our proposed methods. With the exception of ENet [34], all networks are obtained from their official open-source implementation in PyTorch with their respective default configuration (since there is no official Python implementation of ENet, we obtain it from: github.com/gjy3035/enet.pytorch, (accessed on 29 June 2022), which is the most accepted open-source repository of this network). We trained and evaluated all the networks with our proposed CityscapesVid dataset. The training setup is the same for all networks. However, the open-source networks follow a standard training procedure, where the batch images are extracted randomly from the dataset. Our proposal FASSVid, on the other hand, is trained with sequential batches due to the nature of our approach for video segmentation. From the experiments shown in Table 5, we observe that our method obtains the highest FPS rate of all networks by a great margin. Specifically, it is about 1.72× faster than Fast-CNN [29], which is the closest competitor in terms of speed. Additionally, when pretrained with Cityscapes Fine, the mIoU score of FASSVid is comparable to the top result obtained from LEDNet [35]. Note, however, that FASSVid is 7.18× faster and demands about 5.62× less GFLOPs to execute a single run of a 3×1024×2048 input tensor.

**Figure 8:**
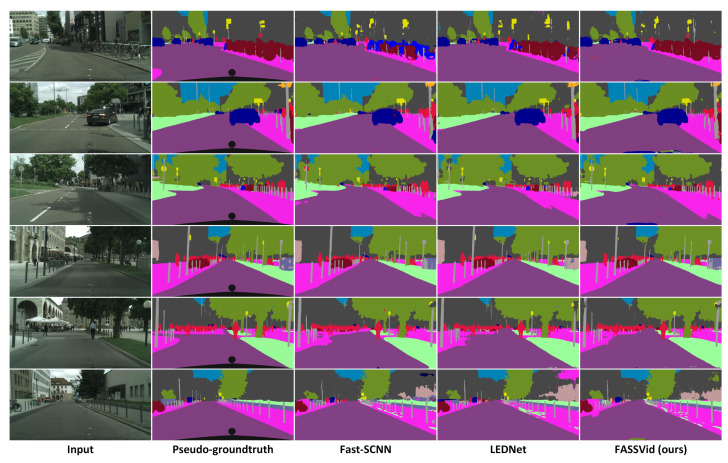
Qualitative results of our proposal compared with two state-of-the-art lightweight networks.

In Figure 8, we show examples of qualitative results of our proposal FASSVid versus LEDNet and Fast-SCNN, which are the closest competitors in terms of mIoU accuracy and FPS, respectively. Note how the results of all networks share common segmentation mistakes, as they are all within the lightweight network category (and in consequence, they tend to be less accurate in general). Qualitatively speaking, Fast-SCNN results are comparatively less accurate than the other two methods. However, the differences between LEDNet and FASSVid cannot always be distinguished visually due to the similar accuracy performance.

### 4.4. Implementation on Jetson Nano

**Figure 9:**
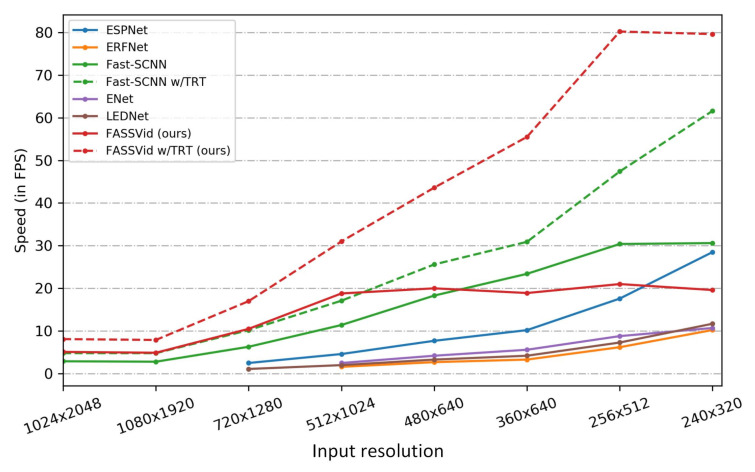
Inference speed in FPS for different input resolutions on Jetson Nano.

We show the speed results of FASSVid implemented on the NVIDIA Jetson Nano (4 GB version), an embedded board capable of performing GPU tasks for low-power applications. For comparison, we also implemented the previously mentioned lightweight networks. The implementation results are shown in Figure 9. As seen in the figure, the standard implementation of our proposal obtains the best results in FPS for high resolution inputs, being only surpassed by Fast-SCNN [29] for low resolution inputs.

We show the optimized implementations using TensorRT [47], an NVIDIA framework for network inference acceleration. Note how TensorRT was only able to optimize our proposal FASSVid and Fast-SCNN. This is due to some dynamic components and deconvolution implementations used by the rest of the networks that are not supported natively by TensorRT. In this scenario, our proposal FASSVid outperforms all other implementations by a great margin for all input resolutions.

## 5. Discussion

In terms of accuracy performance, we observe that, when implemented individually, the improvements in mIoU provided by our proposed modules are rather small. However, when they are unified into a single network, the improvement in mIoU increases considerably in comparison. We believe that our two modules complement each other and better exploit the temporal information when they are combined in this way. On top of that, when our network is trained during more epochs, the accuracy increases further, suggesting that the more it is trained, the more it is able to understand the temporal correlation between the frames of a sequence.

As for the speed performance, the resulting speeds of FASSVid are consistent between the desktop (NVIDIA GTX1080Ti) and the embedded implementation (Jetson Nano). The number of FPSs of our network represents the top results in most of the tests. The only exceptions are in the implementation on Jetson Nano, where, when our model is not optimized by TensorRT [47], it is surpassed by the closest competitor in terms of speed, Fast-SCNN [29]. However, this only occurs at low resolution input images, which lead to highly coarse and inaccurate results, especially for the case of small objects that inevitably tend to disappear during the inference process.

It is worth noticing that most of the state-of-the-art lightweight networks, despite being lightweight in desktop applications, barely run on embedded devices, and when they do, they do it too slowly for many practical applications.

For the most part, the obtained results show that our proposal FASSVid can run with real-time performance even on low-powered embedded systems, such as the Jetson Nano.

## 6. Conclusions

In this paper we presented two key methods to improve semantic video segmentation: DFChangeNet and TAM. DFChangeNet is a small network designed to detect the change information between a pair of frames of the video sequence and to help to enhance certain temporal features coming from the semantic encoder. Similarly, the TAM modules in the decoder serve as an attention mechanism to focus on relevant features from previous predictions of the sequence. In addition, to properly train and test our modules, we proposed *Cityscapes VidSemSeg*, a new dataset based on the official and publicly available video frame data from Cityscapes. The proposed dataset was specifically designed for evaluating the semantic video segmentation task using pseudo-groundtruths generated for each video frame. The combination of our proposals on top of a high-performance encoder result in our network *FASSVid*. FASSVid obtains remarkable inference speed performance with mIoU accuracy comparable to other state-of-the-art lightweight networks. FASSVid is not only fast in desktop implementation, but it also runs substantially faster than other state-of-the-art networks on the NVIDIA Jetson Nano, which demonstrates the effectiveness of the network for low-power embedded applications. As future work, we plan to further exploit the temporal correlation between frames and the previous outputs of the network, by developing multiple loss functions specifically designed for each case. In semantic segmentation tasks, a broad selection criterion is required for the selection of the methods to be used, since these are oriented to solving specific problems, in our case we observe in Table 5, among the compared networks, that there are two that have better results in the mIoU (72.3% and 73.9%) than our proposal (71.0%), but the speed of inference in FPS (18.4 and 16.0, respectively) is very low compared to our results, 114.9 FPS, which we can conclude is due to the proposed method allowing a higher speed of inference which makes it highly appropriate for use in video analysis. While the results are promising for use in video sequences, the proposal will present disadvantages in its use and results when it is intended to be deployed for single images, which do not have a temporal relationship. A major drawback in all semantic segmentation methods is the lack of sufficient pixel-level labelled databases, to address this challenge, ref. [48] proposed a methodology to scale up training sets by synthesizing new training samples and a boundary label relaxation technique that makes training robust concerning annotation noise. This proposal allows making use of specialized models in video processing to generate synthetic training sets; moreover, it can be used to manually improve annotated training sets, so one of the future works is to implement this methodology to scale up our training sets.

## Figures and Tables

**Table 1:** Experiments with the position of FDChangeNet.

Method	GFLOPs	No. Parameters	FPS	mIoU (%)
Baseline	7.9	1.99 M	140.8	68.6
Baseline + FDChangeNet (before C-ASPP)	8.2	2.22 M	123.4	68.4
Baseline + FDChangeNet (after C-ASPP)	8.2	2.22 M	127.4	**69.3**

**Table 2:** Experiments with the number of filters per layer of FDChangeNet.

Method	GFLOPs	No. Parameters	FPS	mIoU (%)
Baseline	7.9	1.99 M	140.8	68.6
Baseline + FDChangeNet (standard version)	8.2	2.22 M	127.4	**69.3**
Baseline + FDChangeNet (light version)	8.0	2.06 M	132.1	67.6

**Table 3:** Experiments with the attention strategy for TAM.

Method	GFLOPs	No. Parameters	FPS	mIoU (%)
*Baseline*	7.9	1.99 M	140.8	68.6
*Baseline + Att*	7.9	1.99 M	101.3	68.0
*Baseline + Att_conv*	8.7	1.99 M	77.8	68.0
*Baseline + Att_conv_all*	8.0	2.0 M	99.7	64.7
*Baseline + Att_conv_dec*	8.0	2.0 M	130.5	**69.1**

**Table 4:** Ablation study of our proposed modules.

Method	GFLOPs	No. Parameters	FPS	mIoU (%)
Baseline	7.9	1.99 M	140.8	68.6
+ FDChangeNet	8.2	2.22 M	127.4	69.3
+ TAM	8.0	2.0 M	130.5	69.1
+ FDChangeNet + TAM	8.2	2.22 M	114.9	**71.0**

**Table 5:** Comparison with other state-of-the-art lightweight networks.

Method	GFLOPs	No. Parameters	FPS	mIoU (%)
ESPNet [32]	13.8	**0.2 M**	42.2	64.8
ERFNet [25]	107.4	2.07 M	13.8	65.6
Fast-SCNN [29]	**7.0**	1.14 M	66.8	66.1
ENet [34]	22.2	0.35 M	18.4	72.3
LEDNet [35]	46.1	0.93 M	16.0	**73.9**
FASSDVid (ours)	8.2	2.22 M	**114.9**	71.0

## Data Availability

Not applicable.
